# Novel approaches for COVID-19 diagnosis and treatment: a nonsystematic review

**DOI:** 10.3906/biy-2105-45

**Published:** 2021-08-30

**Authors:** Şebnem GARİP USTAOĞLU, Hakan KAYGUSUZ, Mehmet Dinçer BİLGİN, Feride SEVERCAN

**Affiliations:** 1 Department of Medical Biochemistry, Faculty of Medicine, Altınbaş University, İstanbul Turkey; 2 Department of Basic Sciences, Faculty of Engineering and Natural Sciences, Altınbaş University, İstanbul Turkey; 3 Sabanci University SUNUM Nanotechnology Research Center, İstanbul Turkey; 4 Department of Biophysics, Faculty of Medicine, Aydın Adnan Menderes University, Aydın Turkey; 5 Department of Biophysics, Faculty of Medicine, Altınbaş University, İstanbul Turkey

**Keywords:** COVID-19, diagnosis, novel methods, infrared spectroscopy, Raman spectroscopy, nanotechnology

## Abstract

Since COVID-19 pandemic has been continuously rising and spreading, several original contributions and review articles on COVID-19 started to appear in the literature. The review articles are mainly focus on the current status of the pandemic along with current status of the corona diagnosis and treatment process. Due to some disadvantages of the currently used methods, the improvement on the novel promising diagnosis and treatment methods of corona virus is very important issue. In this review, after briefly discussing the status of current diagnosis and treatment methods, we present to the scientific community, novel promising methods in the diagnosis and treatment of COVID-19. As with other novel approaches, first, the diagnosis potential of mass spectroscopy and optical spectroscopic methods such as UV/visible, infrared, and Raman spectroscopy coupled with chemometrics will be discussed for the corona virus infected samples based on the relevant literature. In vibrational spectroscopy studies, due to complexity of the data, multivariate analysis methods are also applied to data. The application of multivariate analysis tools that can be used to extract useful information from the data for diagnostic and characterisation purposes is also included in this review. The reviewed methods include hierarchical cluster analysis, principal component analysis, linear and quadratic discriminant analysis, support vector machine algorithm, and one form of neural networks namely deep learning method. Second, novel treatment methods such as photodynamic therapy and the use of nanoparticles in the in-corona virus therapy will be discussed. Finally, the advantages of novel promising diagnosis and treatment methods in COVID-19, over standard methods will be discussed. One of the main aims of this paper is to encourage the scientific community to explore the potential of this novel tools for their use in corona virus characterization, diagnosis, and treatment.

## 1. Introduction

In December 2019, COVID-19 (2019 coronavirus disease) caused by SARS-CoV-2 started to spread from Wuhan City of China, by affecting countries such as Japan, Thailand, and the Republic of Korea in first place (Zhu et al., 2020). In March 2020, it was reported by the World Health Organization (WHO) that COVID-19 has become a “pandemic” to the whole world^1^World Health Organization (WHO) (2020). Advice on the use of masks in the community, during home care and in healthcare settings in the context of the novel coronavirus (2019-nCoV) outbreak: interim guidance [Online]. Website https//www.who.int/publications-detail/advice-on-the-use-of-masks-in-the-communityduring home- care-and-in-healthcare-settings-in-the-context-of-the-novel-coronavirus-(2019-ncov)-outbreak. [accessed March 9th, 2020].. According to WHO data, as of May 15, 2021, more than 161 million people worldwide have been diagnosed with COVID-19, with more than 3.3 million deaths reported^2^World Health Organization (WHO) (2021). WHO Coronavirus (COVID-19) Dashboard [Online]. https://www.who.int. [accessed May 16, 2021].. SARS-CoV-2 has a genetic affinity with the previous coronaviruses named SARS-CoV and MERS-CoV which have previously caused extremely dreadful health threats within two decades (Zhu et al., 2020).

In COVID-19 patients, although it varies from patient to patient, the most common clinical symptoms are sore throat, dry cough, weakness, fever, shortness of breath or breathing difficulties, and body aches. In addition, patients may experience nausea, diarrhoea or runny nose (Xu et al., 2020). In the computerised tomography (CT) scan results of many COVID-19 patients, bilateral, ground-glass opacity with multifocal lung lesions have been reported even in the early stage of SARS-CoV-2 infection (Xu et al., 2020). These ground glass opacities and patchy consolidations can be seen in infected patients without any clinical symptoms (Jin et al., 2020).

Polymerase chain reaction (PCR) and reverse transcriptase (RT)-PCR are common methods for the detection of SARS-CoV-2, since they are accepted as reliable methods for virus detection with high sensitivity and specificity (Li et al., 2020a). Although there are several RT-PCR-based test kits with high accuracy for SARS-CoV-2 detection, there are still various limitations of this application. These limitations include getting the sufficient genetic material with sampling, the high cost of the reagents and equipment and getting results in a long time (Shoaib et al., 2021). Although nucleic acid amplification tests are widely recommended in the diagnosis of COVID-19, it is not possible to ignore the false diagnosis and other serious consequences it may cause due to false negative or false positives results (Li et al., 2020a). The similar features on Chest radiography (CT) scan images of 60%–70% COVID-19 patients were determined (Forouzesh et al., 2020). Ten days after the beginning of the disease, the findings of the infection can be defined in CT results (Dheyab et al., 2021). In addition, findings of infection can be seen in CT results of patients who has negative results in nucleic acid-based tests (Forouzesh et al., 2020). Although CT scanning is cheaper in Turkey than some other countries and more easily applicable in secondary centres compared to PCR test, it is still an expensive imaging system with the need of technical expert. Moreover, it has low specificity in the diagnosis of COVID-19 (Shoaib et al., 2021). Besides these limitations of the technique, the exposure to the radiation is another handicap for the use of this method in the diagnosis (Forouzesh et al., 2020). Serum amounts of immunoglobulin M (IgM) and immunoglobulin G (IgG) antibodies can be used as indicators for infections. The combinations of these two antibodies are used for the determination of the disease severity (Shoaib et al., 2021). According to the Centers for Disease Control and Prevention (CDC) recommendation, serological tests can be used just for the assessment of immunity and determining if a patient has ever been infected but not for clinical diagnostics^3^Centers for Disease Control and Prevention (CDC) (2021). COVID-19 Testing Overview [Online]. Website https://www.cdc.gov/coronavirus/2019-ncov/hcp/testing-overview.html [accessed March 17th, 2021].. Therefore, additional technologies must be developed to address these shortcomings in the early diagnosis of COVID-19.

There is not globally known, proven treatment or drug for COVID-19 disease. On the other hand, there are some medications recommended for treatment in official protocols and guidelines in many countries. Antiprotozoal and antiviral drugs are used in the treatment of COVID-19 patients. In addition to these drugs, antipyretics or NSAIDs, antibiotics, vasopressors and anticoagulants, early blood purification, beta-agonists, and systemic corticosteroids are generally recommended as supportive treatment depending on the prognosis of the disease (Shoaib et al., 2021). As an adjunctive therapy, the beneficial potential of hyperbaric oxygen (HBO) therapy cannot be dismissed. Although the evidence of the beneficial effects of HBO therapy in COVID-19 patients is limited, there are several clinical studies reported that early application of this technique can promote the recovery of the patients (Gorenstein et al. 2020; Zhan et al. 2020; Harrison et al. 2021). The use of chloroquine (CQ) and hydroxychloroquine (HCQ) as antiprotozoal drugs was not supported by WHO due to their failure in treatment and side effects especially hearth diseases when used with other drugs (Saghir et al., 2021). Some of the antiviral drugs (remdesivir, lopinavir, and ritonavir) have not shown better results than the other treatment options used for moderately ill patients, while the others including favipiravir need additional clinical studies (Shoaib et al., 2021). In addition, the drug called REGN-COV2, developed by Regeneron Pharmaceuticals, was approved by the US FDA on November 21, 2020 for the treatment of adult mild-to-moderate COVID-19 patients besides the paediatric patients above 12 years old and 40 kg weight. This drug consists of two monoclonal antibodies, casirivimab, and imdevimab, and is used in all hospitalized COVID-19 patients in the United States. When the drug is used in these patients, it prevents the prognosis of the disease from worsening and the patients to enter intensive care^4^PRNewswire (2021, March 23). Phase 3 Trial Shows REGEN-COV™ (casirivimab with imdevimab) Antibody Cocktail Reduced Hospitalization or Death by 70\% in Non-hospitalized COVID-19 Patients [Online]. Website https://www.prnewswire.com/news-releases/phase-3-trial-shows-regen-cov-casirivimab-with-imdevimab-antibody-cocktail-reduced-hospitalization-or-death-by-70-in-non-hospitalized-covid-19-patients-301253401.html [accessed May 19, 2021].. However, there are limited clinical data with this drug for the treatment of COVID-19 (Weinreich et al., 2021). Thus, the results of the ongoing randomized controlled clinical studies should be closely monitored to determine an optimal treatment strategy in severe COVID-19 patients.

As COVID-19 continues to spread rapidly as a global epidemic around the world, research is ongoing for new diagnostic and therapeutic methods and vaccine development studies that are more effective than those currently used.

This review focuses on the new diagnosis and treatment methods developed as alternatives to current diagnosis and treatment strategies used against SARS-CoV-2. Thus, it will shed light on early diagnosis and effective treatment strategies against possible outbreaks that may occur in the future due to similar coronaviruses. In this review, brief descriptions of the current diagnostic and therapeutic methods were also given with their advantages and disadvantages. There are a great number of reviews related to the action mechanism of SARS-CoV-2, the prognosis, diagnosis, and treatment strategies of COVID-19 since 2020 in the literature. However, this review is a very comprehensive resource that both summarizes currently used techniques and focuses on promising cutting-edge technologies and applications for the diagnosis and treatment of COVID-19.

## 2. Current diagnostic methods

Currently, nucleic acid polymerase chain reaction (PCR) test results are still the gold standard for the diagnosis of COVID-19, and serological tests such as IgG/IgM detection can be used in addition to the nucleic acid test (Ye et al., 2021). Although SARS-CoV-2 can also be detected in various body samples including throat swabs, the upper and lower respiratory tract, serum, urine and feces samples, the use of oropharyngeal swab samples can increase the accuracy of detecting the virus compared to using nasopharyngeal samples, by also reducing the difficulty of sample collection and patient pain. In addition, sputum has been reported to give the highest positive rate with 74.4%−88.9% in the diagnosis of COVID-19 within the first 14 days of the onset of the disease (Zhang et al., 2021).

Currently, there are three main direct and indirect detection methods as Nucleic acid ampliﬁcation test for direct detection, and radiological examination and serological tests as indirect detection methods.

### 2.1. Nucleic acid tests

PCR and RT-PCR are accepted as important nucleic acid-based tests depending on a detection of a specific gene (Shoaib et al., 2021). These methods are commonly used for the determination of SARS-CoV-2, since they are accepted as reliable methods with high sensitivity and specificity for pathogenic virus detection (Li et al., 2020a). RT-PCR which uses RNA template is more recommended than PCR using DNA template (Shen M. et al., 2020). Various RT-PCR-based test kits with 95% accuracy for SARSCoV-2 detection have been developed (Li et al., 2020a). However, to get a reliable PCR-based diagnosis, sufficient sample from the patient, proper handling and storage of these samples and adequate genetic material is needed (Shoaib et al., 2021). In addition, these procedures are expensive techniques that require costly reagents and equipment and give results in a long time. One of the biggest problems in detecting SARS-CoV-2 with these techniques is that safe and stable external positive controls for SARS-CoV (previous coronavirus) are not yet available for SARS-CoV-2 (Shen et al., 2020).

It has been stated that the loop mediated isothermal ampliﬁcation (LAMP) technique, also known as a new ultra-sensitive isothermal nucleic acid amplification-based method, overcomes time-consuming feature and expensiveness of RT-PCR. The advantage of the technique is that it provides results in less than an hour without using expensive reagents or equipment, as well as using a small amount of sample (Tarim et al., 2021). Many studies have initiated clinical applications with RT-LAMP tests with 88.89% sensitivity and 99.00% specificity for the diagnosis of COVID-19 (Hu et al., 2020; Dheyab et al., 2021). However, the requirement for high temperature (mainly 65 °C) and optimization problems related to primers and reaction parameters limit the application of this technique (Shen et al., 2020). 

The “speciﬁc high-sensitivity enzymatic reporter unlocking” method, which is a nucleic acid detection technique based on CRISPR (clustered regularly interspaced short palindromic repeats), combines the LAMP method with the Cas13 enzyme. There is a couple of at-home assays with FDA approval (FDA emergency use authorization) based on CRISPR-Cas13 method for the portable, rapid, and highly sensitive determination of SARS-CoV-2 (Shen et al., 2020). 

### 2.2. Radiological examination

Computerized tomography (CT) of chest is accepted in clinical practice as a reliable method for the diagnosis of COVID-19. The same features including bilateral, ground-glass opacity with multifocal lung lesions are observed on CT scan images of approximately 60% to 77% of COVID-19 cases (Forouzesh et al., 2020). While normal CT findings are observed in more than half of the patients at the first stage of infection (0–2 days), approximately 10 days after the onset of symptoms, the highest lung involvement is reported in CT findings (Dheyab et al., 2021). The limitations of this technique include high cost of the imaging system, requirement for a technical expert and low specificity (25%) in the diagnosis of COVID-19. Although it is reported to have a low specificity, Kovács et al. (2021) stated that CT scans have higher specificity when the low sensitivity of the RT-PCR is considered. It is reported that especially in the patients with negative RT-PCR result with the symptoms of the infection, the use of chest CT has an importance for the diagnosis (Kovács et al., 2021). On the other hand, the CT imaging features seen COVID-19 patients can be overlapped with the characteristic feature of other viral pneumonias (Shoaib et al., 2021). Moreover, especially pregnant women and children need to be avoided from frequent radiation exposure (Forouzesh et al., 2020). 

### 2.3. Serological tests

Although direct diagnostic methods such as specific antigen- or nucleic acid-based methods, are preferred in the diagnosis of COVID-19, indirect detection methods are also widely used in research and clinics. These methods are based on the evaluation of antibodies which are produced as a response when there is an infection in the patient. However, these methods have lower accuracy and specificity compared to direct diagnostic methods (Dheyab et al., 2021).

Serum levels of IgM and IgG which are the antibodies produced in the patient’s body after an infection, can be used as indicators for SARS-CoV-2 infection. The levels of IgM antibody can be used in the early stage of the infection, while IgG antibody levels show variations in the middle or late period of the infection (Tarim et al., 2021). 

Lateral flow IgM and IgG detection for SARS-CoV-2 is one of the point-of-care techniques which is being developed for COVID-19 diagnosis. Lateral flow has 57% clinical sensitivity, 100% specificity and 69% accuracy for IgM, while these percentages are 81 %, 100 %, and 86% for IgG, respectively (Dheyab et al., 2021). 

The enzyme-linked immunospot assay (ELISpot) which is also known as interferon gamma release assay (IGRAs), detects SARS-CoV-2 by measuring the interferon (IFN)-gamma release from T cells in response to the antigens of COVID-19. In the study of Schwarzkopf et al. (2021), it was reported that 78% of PCR-positive patients with undetectable IgG antibodies was successfully detected by using interferon-γ ELISpot assay (Schwarzkopf et al., 2021). However, in another current study, 44% of unexposed healthy patients with an IgG-RBD seronegative, had also a strong T-cell response in the results of COVID-19 ELISpot test. They suggested that this could be caused by a prior exposure to other coronaviruses (Echeverría et al., 2021).

It has been reported that these tests may cause false negative results in patient, as a large population in the community is already exposed to other human coronaviruses with high similarity to SARS-CoV-2 (Lee et al., 2020). According to the recommendations of CDC (Centers for Disease Control and Prevention), serological tests should not be used for clinical diagnostics but just for the assessment of immunity and determining if a patient has ever been infected^1^.

There are also other serological markers which are used for monitoring the severity of the infection and course of the disease in the COVID-19 patients such as interleukins (IL-6, IL-10, and IL-2R), erythrocyte sedimentation rate (ESR), complete blood count (CBC), prothrombin time (PT), and related enzymes of liver, kidney, and heart tissues (Forouzesh et al., 2020).

Due to the limitations of the current methods, additional technologies must be developed to address these shortcomings in the diagnosis of SARS-CoV-2.

## 3. Promising novel diagnosis approaches

The most commonly available methods in diagnostic clinics or hospitals are serological and nucleic acid-based methods. For these purposes, enzyme-linked immunosorbent assay (ELISA) and RT-qPCR methods are widely used. These methods are time-consuming, costly, and not always accurate. In addition, due to shortage of testing facilities the samples have to be transported to available centres which increase the cost and duration of getting the results. There is an urgent need for rapid, inexpensive, sensitive, operator independent diagnostic tool to detect coronavirus 2 [SARS-CoV-2] infections.

### 3.1. Vibrational spectroscopic techniques

Spectroscopic techniques are based on the interaction of electromagnetic radiation with matter. Vibrational spectroscopy mainly includes infrared and Raman spectroscopy. In infrared spectroscopy, infrared radiation interacts with matter. In Raman spectroscopy, the radiation corresponding the electronic energy levels is used but as a low probability event, the transition between the vibration energy levels is detected. These vibrational techniques coupled with chemometric methods are very good candidate to detect viruses including corona virus (COVID-19) (Pahlow et al. 2018; Santos et al. 2020). Vibrational spectroscopy and imaging give valuable molecular information without the need for stains or dyes and with rapid collection all of which simplify and speed up necessary analyses for detection. Other advantages of the techniques will include requirement of less sample size, operator independency and accuracy while not requiring extensive specialized human expertise that stimulate the vibrational techniques to go from bench to bed site.

#### 3.1.1. Infrared spectroscopy

Fourier-transform infrared (FTIR) spectroscopy, especially attenuated total reflection fourier-transform infrared (ATR-FTIR) spectroscopy coupled with multivariate analysis methods is a very good candidate for disease diagnosis including virus-infected samples. When ATR mode is used, very small amount of sample, for example a one drop of bio fluids, is directly put on the top of the crystal and with the improved infrared spectrometers noise free spectral data are collected in a very short time. In addition, FTIR spectroscopy is a rapid, cost-effective, easy to use, nondestructive, operator independent technique. It was also shown that this technique is independent of the mode whether transmission or reflection mode is used (Gok et al, 2016). These properties make this technique a perfect candidate for translation from bench to clinic. Pathological situations induce structural and functional changes in molecules of biological systems. These changes may cause variations in vibrational energy levels which can be detected by infrared spectroscopy (Yonar et al., 2018). Up to now, complex, and large infrared spectral data combined with multivariate analysis methods were successfully employed the diagnosis of different diseases (Severcan and Haris, 2012; Gok et al., 2016; Yonar et al., 2018). Tissue sections, any cytological and histological samples or biofluids can be studied by infrared spectroscopy (Severcan and Haris, 2012).

In a recent review article, the use of ATR-FTIR coupled with multivariate analysis techniques for classification and detection of different viruses such as hepatitis C and B, dengue, zika and chikungunya, were summarized with the aim of using this approach in corona virus detection. The effectiveness of multivariate analysis methods such as principle component analysis (PCA), successive projections algorithm (SPA), genetic algorithm (GA), linear discriminant analysis (LDA), and quadratic discriminant analysis (QDA) were discussed. At the end, it was proposed that ATR-FTIR spectroscopy at mid infrared region can be successfully applied to the detection of COVID-19 virus (Santos et al., 2020). After the pandemic, in last two years, the articles related to the use of FTIR spectroscopy in the diagnosis of COVID-19 has been started to appear in the literature. Barauna and co-workers (2021) first performed laboratory work with spiking of saliva with inactivated COVID-19 virus using ATR-FTIR spectroscopy. It was shown that the virus induced dramatic changes in the nucleic acid bands including RNA. Then, they worked at clinical level with healthy and infected pharyngeal swabs. Swab samples were divided into two categories as negative and positive COVID-19 infection based on symptoms and PCR results (n = 111 negatives and 70 positives). The ATR-FTIR spectra were collected, and then principal component analysis (PCA) was applied to spectra. Following training and validation with the use of 61 negatives and 20 positives samples, by applying genetic algorithm-linear discriminant analysis (GA-LDA) algorithm, 95% sensitivity and of 89% specificity values were obtained. It was reported that it takes only 2 min to get the discrimination of the groups and therefore it will be very useful to use this approach when sudden test is required such as airports, events, or gate controls.

**Figure 1:**
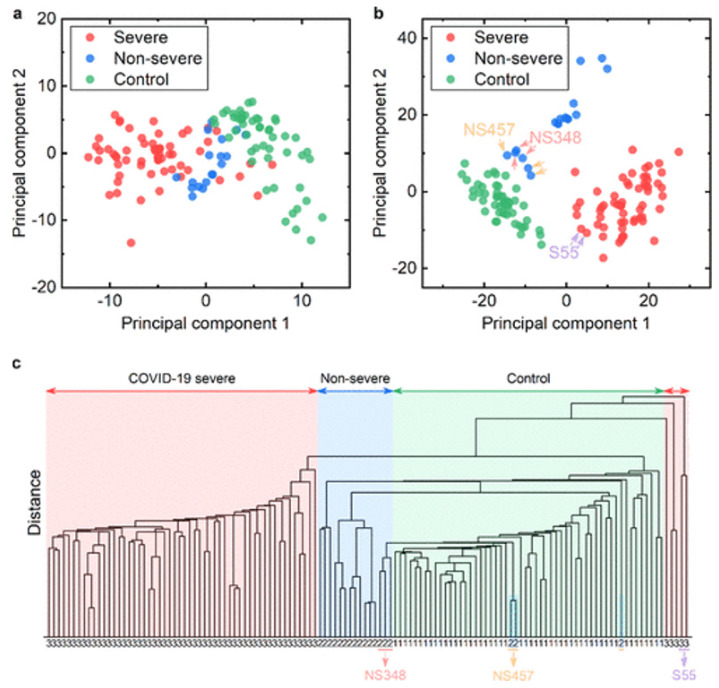
Discrimination among normal controls and nonsevere and severe COVID-19 patients with unsupervised methods. (a,b) PCA score plot using spectral ranges of 1600−1700 cm^−1^ and 1000−1700 cm^−1^, respectively. Spectra from three patients are marked with arrows. NS = nonsevere. S = severe. (c) Hierarchical cluster analysis (1000−1700 cm^−1^). The windows corresponding to the three groups are filled with different colors. 1 = normal controls; 2 = nonsevere COVID-19 patients; 3 = severe COVID-19 patients. [reprinted with permission from Zhang et al., 2021].

In the work of Zhang and co-workers (2021) a total of 115 blood serum samples, 20 of which from healthy donors, and 76 from infected patients, were studied by ATR-FTIR spectroscopy. A total of 41 samples were confirmed as COVID-19, of which 35 were CoV-1 and 6 were CoV-2. The others were from other infectious diseases such as influenza, inflammation. Detailed characterization studies indicated significant changes in the band location of amide A, all the bands (mainly lipid and one weak band of proteins) in the C-H region, protein amide I and II and nucleic acid bands. These changes indicate structural changes in the relevant molecules. Significant changes in the concentration of lipids, proteins and nucleic acids were also observed from the signal intensity values. HCA and PCA unsupervised chemometric tools and a supervised method namely partial least squares discriminant analysis (PLS-DA) were applied to the infrared spectra and based on these spectral variations, the COVID-19 group was successfully separated from the nonsevere infected group and the healthy control group (Figure 1).

#### 3.1.2. Raman spectroscopy and imaging

As a vibrational spectroscopic technique, similar to infrared spectroscopy, Raman spectroscopy and microspectroscopy are also promising techniques in disease diagnosis and screening (Severcan and Haris, 2012). Several papers have been published related to their applications together with different multivariant analysis methods on different human diseases using both cells, tissues, and body fluids (Pahlow et al., 2018). Raman spectroscopy and microspectroscopy are also excellent techiques to study COVID-19 virus.

In a recent review (Saviñon-Flores et al., 2021), mainly basis of surface enhanced Raman scattering (SERS) technique was reported with some examples in identification and characterization of infectious diseases such as influenza (flu) and coronaviruses (CoV), including COVID-19. In the other study, Carlomagno et al. (2021) used SERS technique to the saliva of COVID-19 test positive (n = 30) and negative (n = 38) patients and healthy people as control (N=33) groups. Their results demonstrated the differences in biomolecular content of the three experimental saliva groups. They also applied classification models to Raman spectra such as leave-one-patient-out cross-validation (LOPOCV), support vector machine (SVM), random forest (RF), extreme gradient boosting (XGB), and convolutional neural networks (CNNs). Among these, the most promising results were obtained by CNNs method which revealed as more than 95% accuracy, sensitivity, and specificity. Using deep learning model, 89%–92% accuracy was achieved. The other study also used SERS technique and proposed that, this technique can perform faster detection of COVID-19 than routinely used PCR (Magdy et al., 2020). 

A more detailed study was published recently about the application of Raman spectroscopy coupled with multivariate analysis on COVID-19 diagnosis from serum (Yin et al., 2021). Since it is also very important to detect suspected case which consists of patients with flu symptoms or individuals in tight contact with COVID-19 patients, this group also was included to the experimental groups. A total of 177 serum samples were studied, of which 63 were confirmed COVID-19 patients, 59 suspected cases, and 55 healthy individuals as a control group. The Raman spectra of the COVID-19 infected group were very different than those of other groups. As multivariate analysis machine learning support-vector machine (SVM) method was applied to the Raman spectra to separate the experimental groups. A very good separation was obtained between the groups. The accuracy, sensitivity, and specificity values obtained clearly showed that COVID-19 infected group successfully differentiated from the other groups. Furthermore, in this study external validation was applied using 20 independent samples, 5 of which asymptomatic COVID-19 patients and 5 symptomatic COVID-19 patients, 5 suspected patients, and 5 healthy patients. The model showed that classification results were all correct for independent test dataset. This study suggests that Raman spectroscopy could be a safe and efficient technique for COVID-19 diagnosis and screening.

### 3.2. Mass spectrometry (MS)-based omics technologies

Mass spectrometry (MS)-based omics technologies such as proteomics, glycomics, lipidomics, and metabolomics, has been used for the detection and identification of microbial agents like SARS-CoV-2. MS technologies provide the instant picture of pathogen related changes in the host. SARS-CoV-2 infections and pathogenesis has been searched in the COVID-19 patient’s materials and cellular model systems by MS-based omics technologies (Mahmud and Garrett, 2020). PCR-MS has directly identified all known pathogens in the clinical specimens and detected gene sequences of undiscovered pathogens (Wolk et al., 2012). Matrix-assisted laser desorption/ionization combined with mass spectrometry (MALDI-MS) is an advanced method for the diagnosis of COVID-19 by using minimal specimens, a few reagents, easy to handle protocol and fast data acquisition system but MALDI-MS is currently an expensive tool for many laboratories in the world (Nachtigall et al., 2020). As conclusion, MS-based technologies are the advanced techniques for determination of pathogen outbreaks such as COVID-19.

For the detection of respiratory viruses including SARS-CoV-2, there are other new studies to develop many more original and innovative optical detection methods using absorbance, surface plasmon resonance, localized surface plasmon resonance, fluorescence, and colorimetric techniques in microfluidic devices. These techniques have advantages such as low sample volume, short assay time, low cost, being portable and precise (Dheyab et al., 2021; Tarim et al., 2021). However, these methods are still under development and cannot be used for direct diagnosis of COVID-19.

## 4. Current therapeutic methods

### 4.1. UV-based disinfection technologies

Ultraviolet (UV) technologies have been intensively used for disinfection of hospitals and medical equipment for decades. UV can kill the microorganisms and inactivates viruses including SARS-CoV-2 in all surfaces. SARS-CoV-2 can survive on surfaces for up to 9 days. The effects of UV germicidal irradiation (UVGI, UV-C irradiation at 254 nm) on SARS-CoV-2 depend on culture medium, SARS-CoV-2 concentration, UV-C dose, irradiation time and UV-C absorbance (Biasin et al., 2021). Furthermore, UV-C can cause skin cancer and cataracts in humans. Thus, UVGI protocols for inactivation of SARS-CoV-2 and disinfection of used personal protective equipment are needed to develop by concerning the safety of UV devices (Raeiszadeh and Adeli, 2020). On the other hand, UV-B radiation (up to 20 min) can inactivate the SARS-CoV-2 on surfaces. Also, UV-B enhances the synthesis of vitamin D which leads to protective effects against COVID-19 patients by increasing immunity (Ratnesar-Shumate et al., 2020).

### 4.2. Treatment with antiviral drugs

Over 80 antiviral drugs are approved for treatment of viral infections, including HIV, Ebola, influenza A and B, hepatitis A and C, herpes simplex and human papillomavirus (De Clercq and Li, 2016). After the outbreak of COVID-19 pandemic, several antiviral agents have been used in order to treat the disease, and the reports are numerous. Several review articles are already reviewed this topic again repeatedly, and in this part the findings of some of these review articles as well as original reports have been discussed. 

There are several reports on favipiravir treatment. These include a study in China with 80 patients where they received 1600 mg twice on the first day and 600 mg twice a day on between 2nd–14th days with interferon-α aerosol inhalation (Cai et al., 2020). Results were promising as favipiravir treatment showed less adverse effects and better therapeutic effects. Several systematic reviews also show the use of favipiravir treatment is a good option thanks to rapid viral clearance and fast clinical improvement (Joshi et al., 2021). Currently, favipiravir is among the antiviral agents used in COVID-19 treatment worldwide. The mechanism of favipiravir is the substitution with purine nucleotides in viral RNA after intracellular reactions (Tarighi et al., 2021).

Darunavir is a nonpeptidyl HIV-1 protease inhibitor, which is used for treatment of HIV infections by selectively inhibiting Gag-Pol polyprotein cleavage (Tarighi et al., 2021). Initial predictory studies on several commercially available studies, including darunavir, reported as a potential candidate (Beck et al., 2020), however the study by De Meyer et al. showed no antiviral activity of darunavir against SARS-CoV-2 (De Meyer et al., 2020). On the other hand, several studies reported the use of darunavir along with cobicistat (Valentina et al., 2020) and umifenovir (Costanzo et al., 2020).

Neuraminidase inhibitor oseltamivir is used for influenza A and B viruses, where it shows its activity by blocking neuraminidase which plays a role in viral entry to host cells as well as viral release from infected cells (Tarighi et al., 2021). Although some initial studies reported that oseltamivir has positive in treatment of COVID-19 patients, recent reports indicated its ineffectivity for COVID-19 patients (Tan et al., 2020). Another antiviral for influenza treatment, umifenovir, was also reported a little benefit for the mild/moderate COVID-19 patients when applied at 100 mg dose (Li et al., 2020b) but another study at 200 mg three times daily, significantly shows improvements in clinical and laboratory findings (Nojomi et al., 2020), indicating the need for further studies with larger sample sizes.

Remdesivir is a RNA polymerase inhibitor which is shown as an effective therapeutic agent for treatment of COVID-19 (Wang et al., 2020) and it is the first FDA approved treatment for COVID-19^5^FDA (2020). FDA Approves First Treatment for COVID-19 [online]. Website https://www.fda.gov/news-events/press-announcements/fda-approves-first-treatment-covid-19 [accessed April 27, 2021].. Its antiviral effects are active by interfering viral replication inside the host cell by forming a complex with SARS-CoV-2 NSP12 RNA dependent RNA polymerase (Rezagholizadeh et al., 2021). According to a recent systematic review, remdesivir could improve 28-day recovery rate as well as invasive mechanical ventilation & extracorporeal membrane oxygenation (Rezagholizadeh et al., 2021).

Other employed antiviral drugs include lopinavir, atazanavir, emtricitabine and sofosbuvir (Frediansyah et al., 2021). Among the antiviral drugs, remdesivir, lopinavir and ritonavir have not shown better results than the other treatment options used for moderately ill patients according to the one of the largest randomized controlled trials to date, WHO solidarity trials (Shoaib et al., 2021).

REGN-COV2; antibody cocktail drug involves two monoclonal antibodies namely casirivimab and imdevimab. These antibodies used in the drug REGN-COV2 for the treatment of COVID-19 target different parts of the spike protein of the SARS-CoV-2 virus and bind tightly and noncompetitively to this protein. This prevents the virus’s ability to infect healthy cells (Weinreich et al., 2021). REGEN-COV2 drug has not been approved but has been authorized for emergency use by FDA. Regeneron reported that phase III trial of REGEN-COV2 antibody cocktail decreased hospitalization and/or death by 70% in nonhospitalized COVID-19 patients as well as shortened the duration of symptoms by 4 days^4^.

## 5. Novel promising therapeutic methods for COVID-19

Novel promising therapeutics which are reviewed here, are not yet authorized, or approved from FDA. Thus, accepted treatment methods should be used as a priority in COVID-19 patients with the recommendations of the national or international guidelines.

### 5.1. Photodynamic therapy: alternative treatment modality against COVID-19

Photodynamic therapy (PDT) is a treatment modality that uses the photosensitizer activated in the presence of visible or near infrared light, and molecular oxygen to produce reactive oxygen species (ROS) such as singlet oxygen (1O2) and/or superoxide anion, hydroxyl radicals, and hydrogen peroxide that eventually target cell death by apoptosis, autophagy, or necrosis without or minimal injuring the adjoining tissues (Ozlem-Caliskan and Bilgin, 2018; Kessel, 2019). PDT is approved for the treatment of certain cancers, precancerous, and noncancerous disorders (Agostinis et al., 2011). Additionally, this approach has been reported to be effective for various types of microorganisms such as bacteria (Gram-positive and Gram-negative), fungi, parasites, and viruses (Jori et al., 2006).

Numerous classifications of photodynamic therapy, for instance photodynamic inactivation, photodynamic antimicrobial chemotherapy, antimicrobial PDT, antibacterial PDT, antifungal PDT, antiviral PDT have been used for treatment of bacterial, fungal, parasitic, and viral disorders (Wiehe et al., 2019).

The main advantages of antimicrobial PDT are a multi-target therapeutic approach and the absence of the development of resistance mechanisms. These properties are depending on the photodynamic action and affected targets on the microorganisms (Almeida et al., 2020). PDT has been applied successfully to inactivate viruses and bacteriophages in the beginning of 20th century. First clinical human trial used of PDT against viruses was conducted in the early 1970s (Felber et al., 1973). However, antiviral PDT has not been accepted easily because of the limitations related to structure of photosensitizers, target specificity, tissue penetration ability. Recently, photoinactivation of viruses such as HIV, HSV, and hepatitis virus families, in the blood and blood products is the main use of antiviral PDT. Methylene blue, riboflavin, curcumin, fullerenes perylene quinones, porphyrin, and porphyrinoids are the photosensitizers for antiviral PDT (Wiehe et al., 2019) but the efficiency of the treatment is significantly dependent on the type of virus and where the PDT applied. Therefore, the use of nanoparticle technologies might improve the delivery, bioavailability, selectivity, and functionality of current photosensitizers while decreasing the side effects.

**Figure 2:**
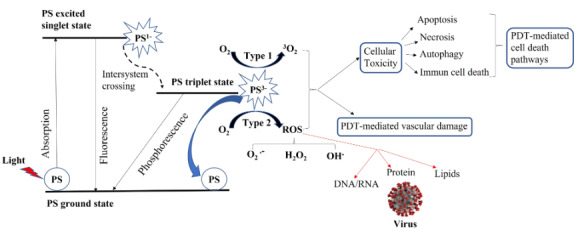
Action mechanism of photodynamic therapy (modified from Jablonski diagram). PS, ground state of the photosensitizer; PS^1^*, excited singlet state of photosensitizer; PS^3^*, excited triplet state of photosensitizer; ROS, reactive oxygen species; O_2_, ground state of oxygen; ^1^O_2_, singlet oxygen; O_2_^−.^, superoxide; HO^.^, hydroxyl radicals; H_2_O_2_, hydrogen peroxide.

PDT is a two-stage procedure. After the administration of a light sensitive photosensitizer, target cells/tissues are irradiated with an appropriate wavelength of visible or near infrared light, which activates a photosensitizer localized in the target tissue in the presence of molecular oxygen (Ozlem-Caliskan and Bilgin, 2018). The action mechanism of PDT is diagrammatically explained in Figure 2. Briefly, under the light irradiation, the photosensitizer in its ground state absorbs photons of appropriate wavelength and photosensitizer is excited to singlet state (PS1*). Excited photosensitizer is very unstable and emits this excess energy as fluorescence and/or heat. Alternatively, an excited photosensitizer may form a more stable excited triplet state (PS3*) via an intersystem crossing. Subsequently, excess energy of the photosensitizer in triplet state can be lost by the emission of phosphorescence, generation of heat or attending of the biochemical reactions. In the presence of biological substrate and molecular oxygen the photosensitizer in triplet state can decay to ground state via type I and type II reactions leading to the formation of singlet oxygen and reactive oxygen species (O2.-, HO., H2O2) (Grossweiner et al., 2005). During the sensitization of photosensitizers, Type I and Type II processes occur simultaneously and compete with each other. The singlet oxygen plays a more important role in PDT for cancers, but reactive oxygen species (ROS) is more effective for the inactivation of microorganism via PDT. Highly reactive oxygen species lead to cellular toxicity in the process of apoptosis, necrosis, autophagy and immune cell death. PDT mediated cell death pathways depend on the location of photosensitizers in the target cells such as mitochondria, cell membrane, lysosomes or endoplasmic reticulum. Singlet oxygen and ROS can also cause destruction of cancer vasculature and produces an acute inflammatory response that attracts leukocytes such as dendritic cells and neutrophils. At the end, these oxygen species cause cellular photo-damage which can activate a repair mechanism or lead to cell death when the damage is beyond repair (Grossweiner et al., 2005). In antiviral PDT, ROS are mainly generated and reacted with nucleic acids (DNA or RNA), virus proteins and viral lipids (Wiehe et al., 2019). Then, virus inactivation is formed that inhibiting virus binding with the host and ultimately ending the virus life cycle. 

The most used medical application of antiviral PDT is decontamination of blood products by mostly using riboflavin, methylene blue (MB) and amotoselen (Schlenke, 2014). Aminolevulinic acid, hematoporphyrin derivatives and MB mediated PDT are predominantly used for the treatment of both local Herpes simplex virus infections and Human papillomavirus infections (viral warts, condylomata accuminata). MB, a positively charge molecule, can cross the viral envelop and intercalate with viral nucleic acid which are negatively charged. Porphyrins have high affinity to viral proteins and lipids. Thus, MB and Radachlorin mediated PDT inhibits SARS-CoV-2 in vitro (Svyatchenko et al., 2021). MB has ability to inhibit the SARS-CoV-2 spike protein and its receptor ACE2 which is crucial in inactivating the virus. MB mediated PDT has been used in dental disorders for decades, but PDT was clinically used for oral manifestation of COVID-19 recently. MB mediated PDT with photobiomodulation therapy, known as low level laser therapy, were shown effectiveness in the management of COVID-19 related orofacial lesions. The combination of both methods causes for removing the viruses, enhancing tissue oxygenation and reducing or inhibiting cytokine storm. Therefore, improvements in tissue repair and pain reduction have been observed in these patients (Teixeira et al., 2021).

PDT can be used for the treatment of COVID-19 infections in the respiratory tract. An extracorporeally-illuminated PDT was used against bacterial pneumonia by using extracorporeal illumination at 780 nm light with nebulized indocyanine green (Kassab et al., 2019). PDT protocols in respiratory tract are still investigated for clinical patients (Dias et al., 2020). Thus, PDT against COVID-19 may reduce the microbial load in the respiratory tract in near future. Also, if a photosensitizer like indocyanine green can deliver to the lungs, it may be irradiated by a laser. At the end, PDT generates singlet oxygen and ROS to initiate the virus inactivation.

The photosensitizer, light, and molecular oxygen play an important role for PDT against viruses such as COVID-19. Depending on the advancing technologies, various photosensitizers in nanoparticles and their physicochemical reactions of the target cells can be used for increasing the therapeutic efficacy of viral PDT. The amount oxygen consumption is directly related to light dose and source of light. Therefore, oxygen monitoring within the target cells or tissue may also crucial for PDT. Although there are significant challenges for PDT, it has a potential use for the management of COVID-19.

### 5.2. Nanotechnology based methods

Nanotechnology can play an important role in COVID-19 treatment, as previous antiviral studies utilizing nanotechnology showed (Tang et al., 2021). The most important advantage of nanomaterials antiviral research is the similarity of size where the size of SARS-CoV-2 (60−140 nm) (Zhu et al., 2020) is similar to FDA approved nanomaterials (Tang et al., 2021). Other advantages include the use of nanomaterials in delivery of drugs, enhancing the stability of vaccines (Tang et al., 2021). Besides its advantages in diagnosis (Vahedifard and Chakravarthy, 2021), applications in treatment are currently being researched. When it comes to antiviral treatment, it should be noted that all the antiviral formulations used for other viral infections are being challenged for treatment of COVID-19, by the limited intracellular intake/uptake of the molecules (Ansari et al., 2020), therefore nanoformulations can be very effective for targeting the viruses and targeted delivery of the antiviral molecule (Lembo et al., 2018). Potential use of nanotechnology in the treatment is the inhibition of virus-cell interaction, membrane fusion, cell internalization, transcription and translation, and activating intracellular mechanisms (Cardoso et al., 2020; Mainardes and Diedrich, 2020).

#### 5.2.1. Inorganic nanoparticles

Inorganic nanoparticles forms a large family of materials, including metal oxide nanoparticles (Kalaycıoğlu et al., 2020, Kaygusuz and Erim, 2020), silica nanoparticles (Farjadian et al., 2019) and metallic nanoparticles (Zhang et al., 2016). Some nanomaterials such as silver nanoparticles have direct effect as virucidal activity (Zhou et al., 2021). The efficiency of silver nanoparticles as antiviral agents is well studied for viruses such as HIV and HSV and even practical applications are proposed (Mohammed Fayaz et al., 2012). Gold nanoparticles are also promising thanks to the good surface chemistry properties for health applications and other properties (Rashidzadeh et al., 2021). The antiviral mechanism of gold nanoparticles is including the blocking of gp120 attachment with CD4 for viral entry inhibition and the antiviral mechanism of silver nanoparticles is corresponding to viral entry inhibition, attachment or replication (Maduray and Parboosing, 2020). Other metallic nanoparticles with antiviral activity include zinc, iron and copper. Other advantages of metallic nanoparticles are ease of manufacture, surface modification and size tuning.

As for metal oxides, they are mostly used for detection purposes, but the treatment reports are also present. When nanomaterials show enzyme-like characteristics, they are called nanozymes (Wu et al., 2019). For example iron oxide nanoparticles show enzyme-like activity by peroxidase and catalase activities and already reported inactivation of influenza (Gao et al., 2017; Qin et al., 2019). Other example to metal oxide nanoparticles with antiviral activities is TiO2 (Akhtar et al., 2019).

Inorganic nanoparticles have several limitations for effective delivery and avoiding toxicity. These parameters include surface properties, size, charge, and shape of the nanoparticles (Vahedifard and Chakravarthy, 2021). By the means of targeted delivery, one option is the intranasal delivery of the nanoparticles and other options are targeted drug delivery and specific small interfering RNA (siRNA) delivery (Itani et al., 2020). Based on the approach and the aim, the design of the surface properties of the inorganic nanoparticle should be precisely carried out.

#### 5.2.2. Lipid based nanotechnology

Liposomes are spherical vesicles consisting of at least one lipid bilayer where the size can be in rage of 15 to 1000 nm (Chakravarty and Vora, 2020) and can be described as most common targeted drug delivery agents (Sercombe et al., 2015). Drug delivery formulations based on liposomes include intracellular functional protein delivery (Chatin et al., 2015), delivery of anti-cancer drugs (Olusanya et al., 2018), gene delivery (Balazs and Godbey, 2011), vaccine delivery (Schwendener, 2014), delivery of antibiotics (Gonzalez Gomez and Hosseinidoust, 2020) as well as antiviral delivery (Düzgüneş et al., 2005). 

Since various water soluble molecules such as proteins, peptides, carbohydrates or nucleic acids can be entrapped within the aqueous inner shell of liposomes, and lipophilic compounds are entrapped within the lipid bilayer, the liposomes are very advantageous in vaccine delivery systems (Schwendener, 2014). After the outbreak of COVID-19 pandemic, several vaccine formulations have been developed and some of them (BNT162b2, mRNA1273) already contains lipid nanoparticles (Milane and Amiji, 2021) to deliver mRNA. mRNA1273 by Moderna contains proprietary ionic lipid SM-102^6^Moderna (2020). mRNA-1273-P301-Protocol [online]. Website https://www.modernatx.com/sites/default/files/mRNA-1273-P301-Protocol.pdf [accessed May 8, 2021].  and BNT162b2 by Pfizer/BioNTech contains the lipid mixture ((4-hydroxybutyl)azanediyl)bis(hexane-6,1-diyl)bis(2-hexyldecanoate), 2-[(polyethylene glycol)-2000]-N,N-ditetradecylacetamide, 1,2-distearoyl-sn-glycero-3-phosphocholine, and cholesterol^7^Medicines and Healthcare Products Regulatory Agency (MHRA) (2021). Information for Healthcare Professionals on Pfizer/BioNTech COVID-19 vaccine [online]. Website https://www.gov.uk/government/publications/regulatory-approval-of-pfizer-biontech-vaccine-for-covid-19/information-for-healthcare-professionals-on-pfizerbiontech-covid-19-vaccine [accessed May 8, 2021]. ^7^. On the other hand, the stability of lipid based nanoparticles can be a drawback. In order to improve the chemical and physical stability of lipid bilayer systems, a recent study utilizes immobilization in a crystalline zeolitic-imidazole framework exoskeleton (Herbert et al., 2021).

Another study by McKay et al. reports the development of self-amplifying RNA encoding the SARS-CoV-2 spike protein encapsulated lipid nanoparticles as a vaccine where the results are very promising with high cellular responses (McKay et al., 2020).

To the best of our knowledge, currently, there are no specific and verified antiviral drugs for SARS-CoV-2, however there are hypotheses for the design of Tat-peptide conjugated repurposed drug by lipid based nano-delivery (Ansari et al., 2020). Another study by Wang et al. proposes the use of low-water solubility anthelminthic drug niclosamide delivery by lipid nanoparticles for inhibition of SARS-CoV-2 replication (Wang et al., 2021). 

## 6. Conclusion

From the influenza pandemic (H1N1) in 1918 to the new coronavirus (SARS-CoV-2) pandemic that emerged in 2019, a suitable technique for the early detection of pathogenic viruses has not yet been developed. The current diagnostic methods have various limitations including the cost of the equipment and reagents, need of technical expert, low specificity to determine SARS-CoV-2 and still they may cause false negative or false positives results. Moreover, although there is not yet a proven and globally known treatment method for COVID-19 disease caused by the SARS-CoV-2 virus, there are medications recommended by official protocols for treatment in different countries. This review focuses on the current diagnosis and treatment strategies used against SARS-CoV-2, and new diagnosis and treatment methods developed as alternatives to current ones. Thus, it will shed light on early diagnosis and effective treatment strategies against possible outbreaks that may occur in the future due to similar coronaviruses. 
